# Synovial fluid and synovial membrane mesenchymal stem cells: latest discoveries and therapeutic perspectives

**DOI:** 10.1186/scrt501

**Published:** 2014-10-03

**Authors:** Eduardo Branco de Sousa, Priscila Ladeira Casado, Vivaldo Moura Neto, Maria Eugenia Leite Duarte, Diego Pinheiro Aguiar

**Affiliations:** Program of Cell and Developmental Biology, Institute of Biomedical Sciences, Federal University of Rio de Janeiro, Avenida Carlos Chagas, 373 Bloco F, Rio de Janeiro, RJ CEP 21941-902 Brazil; Research Division, National Institute of Orthopaedics and Traumatology, Avenida Brasil, 500 Anexo IV – 1° andar, Rio de Janeiro, RJ CEP 20940-070 Brazil

## Abstract

Mesenchymal stem cells (MSCs) have the ability to differentiate into osteoblasts, chondroblasts, adipocytes, and even myoblasts. Most studies have focused on finding MSCs in different parts of the body for medical treatment. Every joint structure, including bone, joint fat, articular cartilage, and synovium, potentially contains resident MSCs. Recently, a progenitor cell population has been found in synovial fluid and showed similarities with both bone marrow and synovial membrane MSCs. Synovial fluid MSCs have been studied in healthy persons and osteoarthritic patients in order to explore its potential for treatment of some orthopedic disorders. Here, we briefly review the current knowledge on synovial fluid MSCs, their origin, relation to some orthopedic diseases, and future applications.

## Introduction

Osteochondral defects may progress to osteoarthritis (OA), causing pain and disability [1]. Many attempts have been made to treat cartilage defects and prevent joint degeneration. Surgical options include microfracture, autologous chondrocyte implantantion, and osteochondral autograft or allograft, but no treatment has been able to restore the natural structure and composition of cartilage [2]. Thus, unfortunately, until today, no treatment has been proved to stabilize, reverse, or prevent OA development.

In the last decades, stem cells have proven to be useful in tissue regeneration and treatment of many diseases. Mesenchymal stem cells (MSCs) have been identified in both healthy and diseased cartilage, and their potential in cartilage regeneration has been studied *in vivo* [3, 4].

Recent findings have led to a discussion that synovial fluid (SF) fibroblasts displayed activated phenotypes, which mediated cartilage destruction through inflammation in the synovium [5]. SF fibroblasts have been demonstrated to be closely related in phenotype to bone marrow (BM) MSCs [6]. This suggests that normal SF has a resident MSC population that increases during OA [7]. MSCs were also found in higher number in SF from knees after meniscal injury than in normal ones [8]. However, the origin and role of these SF MSCs have not yet been determined. The goal of this review is to provide the current knowledge on SF MSCs, their origin, and relation to other joint tissues in normal and arthritic conditions.

## Synovial membrane, synovial fluid, and synovial fluid mesenchymal stem cells

The synovial membrane (SM) is a specialized mesenchymal tissue lining the spaces of diarthrodial joints, bursae, and tendon sheaths [9]. SM includes two layers: the intima inner layer, composed of one or two sheets of macrophages or fibroblast-like synoviocytes, and the subintima outer layer, composed of two to three layers of synoviocytes lying over loose connective tissue rich in fibroblasts, secreting collagen, and other extracellular matrix proteins. The subintima layer has few macrophages and lymphocytes, fat cells, and blood vessels, which provide nutrients to the SM and the adjacent avascular cartilage [9–11].

Cells from the SM intimal layer secrete the SF, which provides articular cartilage lubrication, chondrocyte activity, and nutrition. The synovial intima is composed of two different cell types: type A and B synoviocytes [12]. Type A and B synoviocytes present cell surface markers that identify them as coming from macrophage and fibroblast lineages, respectively [13].

Type A synoviocytes (synovial macrophages) stand round the upper part of synovial lining, whose surface is covered by microvilli and microplicae, like typical macrophage structures [12]. These cells proliferate in inflammatory conditions. Type A SM cells are positive for CD163 and CD68, but not for CD14^+/lo^, and present non-specific esterase activity [9]. Macrophages are not only in the intimal but also in the subintimal layers of SM, deriving from circulating monocytes originated from the BM [9]. Synovial intimal fibroblasts express the surface marker CD55, which is used to distinguish them from synovial macrophages [9]. Under pathological conditions, synovial macrophages may contribute to cartilage destruction due to prolonged production of pro-inflammatory cytokines or through the formation of osteophytes by the release of the growth factors transforming growth factor-beta (TGF-β) 3 and bone morphogenetic protein (BMP)-2 and BMP-4 [14, 15].

Type B synoviocytes (synovial fibroblasts), which are fibroblast-like cells, express the class II major histocompatibility molecule, which confers a key role in antigen presentation in early phases of immune responses in the SM. Type B synoviocytes are found further from the synovial lining and produce mainly the glycosaminoglycan hyaluronic acid (HA), one of the main constituents of cartilage extracellular matrix, involved with cell signaling by binding to cell receptor CD44 [11]. In this regard, SM intimal type B synoviocytes possess high uridine diphosphate (UDP) glucose dehydrogenase (UDPGD) activity, which converts UDP-glucose to UDP-glucuronate, essential for HA synthesis [9]. In addition, these SM cells produce lubricin, which is responsible for protecting the surface of articular cartilage [12].

In normal conditions, HA and lubricin are not filtered and play a role in joint lubrication due to HA’s high molecular weight, which provides SF viscosity [12]. Lubricin helps in joint lubrication, thus reducing pathologic deposition of proteins over the articular cartilage surface [12, 16, 17], and small molecules such as growth factors and cytokines instead easily diffuse in the SF [12]. These results showed that SM also acts as a semipermeable membrane that controls molecules traffic and the composition of the SF [2, 12].

## Characterization of synovial fluid mesenchymal stem cells

MSCs have been defined following the studies by Friedenstein and colleagues [18] and Pittenger and colleagues [19] as BM cell components of non-hematopoietic origin. In this regard, BM spindle-shaped adherent cells with heterogeneous appearance were described. In the 1980s, other investigators established that those cells were multipotent, able to differentiate in osteoblasts, chondroblasts, adipocytes, and, in certain conditions, myoblasts [17, 20]. MSCs were also identified in several organs as cells that have the function of replacing local cells lost in physiological turnover or repairing and regenerating injured tissues [21].

Microscopic analysis revealed cell aggregates entrapped in fibrin, typical of inflammatory SF. Intact pieces of synovium have been documented, but synovial cells were not well characterized *in vitro*. Some findings demonstrated the presence of clonogenic and multipotent MSCs in the SF of both young animals and human joints not affected by arthritis. Since these fragments are significantly hyperplastic, they seem to be a result of avulsion from weakened synovium because of a lack of nutrients [3]. These fragments are probably not related to the origin of SF MSCs.

According to the International Society for Cytotherapy, MSCs must be able to adhere to plastic material and expand when cultured *in vitro*. In addition, MSCs must express the surface markers CD73, CD90, and CD105 and be negative for the expression of CD45, CD34, CD14, CD11b, CD79α, CD19, and HLA-DR surface molecules. Moreover, MSCs must show the ability to differentiate into three lineages of mesenchymal cells: osteoblasts, chondroblasts, and adipocytes [22].

Comparison among MSCs isolated from BM and adipose tissue (AT) and SM or synovium showed that these cells do not express hematopoietic markers, such as CD11b [22, 23], CD14 [23, 24], CD19 [22],CD31 [23, 24], CD34 [22, 23], CD45 [22–25], CD79α [22], CD117 [23], and HLA-DR [22], but must have the expression of CD13 [24], CD29 [25], CD40 [26], CD44 [24, 25], CD49e [27], CD73 [23, 24], CD90 [24, 25], CD105 [23–25], CD147 [25], CD166 [23, 24], Notch1 [27], and STRO-1 [23] on their surfaces (Table 1). Although the cell marker CD271 is expressed in AT and BM MSCs, its expression seems to be negative in healthy SM MSCs [24]. However, CD271 was demonstrated to be expressed in the SM of patients with OA [28].

**Table 1:** **Mesenchymal stem cell markers for cells derived from synovial membrane, cartilage, fat pad, bone marrow, and synovial fluid**

Tissue	Positive markers	Negative markers	References
Synovial membrane	CD90, CD105, CD147, and CD44	CD34, CD45, CD117, and CD31	De Bari *et al*. [17] (2001), Sakaguchi *et al*. [29] (2005)
Cartilage	CD49e, Notch1, CD90, and STRO-1 antigen		Williams *et al*. [27] (2010), Alsalameh *et al*. [30] (2004)
Fat pad	CD13, CD29, CD44, CD90, and CD105	CD34, CD56, CD271, and STRO1	Khan *et al*. [31] (2012)
Bone marrow	CD13, CD29, CD44, CD90, and CD105	CD34 and CD45	Barry and Murphy [32] (2013)
Synovial fluid	CD40, CD44, CD73, CD90, CD 105, and CD 166	CD11b, CD34, CD45, and CD271	Boeuf and Richter [24] (2010), Krawetz *et al*. [26] (2012)

SF MSCs show high expression of hialuronan receptors CD44 and UDPGD, required for hialuronan synthesis. During synovial joint development, prior to cavitation, CD44 is expressed in the interzone and the articular surfaces. Conversely, UDPG activity is increased in the articular surfaces but is lowered in the interzone. HA free and bound are found at this time in the interzone. After cavitation, synovium and articular surfaces bind CD44 to HA. This process facilitates tissue separation and helps create a functional joint cavity [33]. In cultures enriched with CD90 cells, levels of chondrogenesis were higher than in culture fractions depleted of CD90 [26]. The CD90 receptor was demonstrated to interact with integrins, tyrosine kinases, growth factors, and cytokines, promoting downstream cellular events such as adhesion, apoptosis, proliferation, and migration. However, the heterogeneity of synovial cells has not been well described [26].

Functional studies showed that SF MSCs were distinct from BM MSCs; SF MSCs form a pool of highly clonogenic cells with chondrogenic potential, whereas BM MSCs are very heterogeneous. This fact suggested that SF MSCs were not derived from BM MSCs but from adjacent synovium [7]. This corroborates the findings from another study, which showed that SF MSCs seemed to be closer to synovium MSCs than to BM MSCs [34]. Another study revealed that intra-articular bleeding soon after anterior cruciate ligament rupture leads to a shift of SF MSCs by expression of cytokines and chemokines that recruit MSCs from elsewhere. It showed that the number of SF MSCs increased after the lesion, suggesting that SF MSCs originated neither from BM nor from circulating MSCs but probably from the synovium or cartilage [35, 36].

## Therapeutic potential for synovial membrane stem cells

Synovial inflammation is present in early OA and is possibly the trigger of the cascades leading to articular destruction but may also be the focus of repairing responses from progenitor cells [32]. In patients with OA, there is a decrease on the cartilage extracellular matrix components type II collagen fibers and aggrecan proteoglycan, the latter of which is formed by HA associated with distinct sulfated glycosaminoglycans, the main one of which is chondroitin sulfate. This condition leads to a reduction in the absorption of mechanical forces [16]. Moreover, SF lubricin decreases in OA, contributing to lower articular cartilage lubrication [37]. In addition, higher levels of vascular endothelial growth factor were demonstrated in SF of patients with OA versus rheumatoid arthritis, indicating that angiogenesis might play a role in cartilage degeneration. Also, the activation of pro-inflammatory cytokines, such as IL-6, IL-8, interferon-gamma, and monocyte chemoattractant protein-1, of human chondrocytes by OA patient SF supports the pro-inflammatory process in the development of OA [38]. Moreover, elevated IL-15 levels are found in the synovium of patients with early knee OA, providing evidence of activation of innate immunity within SM [39].

Immature articular cartilage contains a population of progenitor cells responsible for its appositional growth. Notch 1 is present in the chondrocytes of the surface zone articular cartilage, determining the proliferation of these cells. Thus, Notch-1 signaling has been associated with healthy cartilage progenitors [32, 40]. Overexpression of Notch-1, Notch-2, RbpJ, and Hes1 has been observed in chondrocyte differentiation [41]. Besides, Notch-1-positive cells were found in greater numbers in cartilage clusters from patients with OA than in control experiments [32]. Cartilage clusters are a typical phenotype of OA and may result from dedifferentiation and proliferation of resident chondrocytes, although migration of progenitor cells cannot be discarded [32].

SM has an intrinsic ability of regeneration given its recovery after sinovectomy [35], suggesting that SM could act as a cell source for cartilage repair [25]. Synovial chondromatosis is a rare proliferative condition of unknown etiology. It evoked a possible role of SM MSCs in the production of multiple intrasynovial cartilaginous nodules [42]. MSCs isolated from the SM, called SM-derived stem cells [17], or synovial mesenchymal progenitor cells [43] were shown to share the same phenotypic and functional properties of BM MSCs [44]. The presence of MSCs in the synovial lining leads to questions on their origin. They could have been recruited from blood that penetrates synovial tissue or originated from BM that connects to intra-articular space. The possible role of MSCs in synovial lining is related to healing potential of tissues originated from the mesoderm. Furthermore, their cells may be involved in early stages of osteoarticular diseases. BM MSCs and SM MSCs express different genes, including activin A, which is upregulated in BM MSCs [44]. Unlike BM MSCs, MSCs derived from SM maintain proliferation rate and colony-forming potential regardless of the age of the patient [43, 45].

SM MSCs have high self-renewal ability and multipotentiality inherent to single cells. Single cell-derived MSCs become heterogenous during expression. Non-clonal plastic adherent synovial MSCs consist of a uniform cell population [46]. Koga and colleagues [47] demonstrated that transplanted synovium-derived MSCs were altered according to the microenvironments in rabbits.

Cells derived from human synovium were shown to have the greatest chondrogenesis potential among the mesenchymal tissue-derived cells, representing a possible source for cartilage repair. Synovium and AT MSCs were demonstrated to be superior in terms of adipogenesis. In addition, MSCs derived from BM, synovium, or periosteum were shown to be superior in osteogenesis [29]. Studies revealed that synovial MSCs precultured with autologous human serum were able to differentiate into chondrocytes *in vitro* but that their chondrogenic potential was lower than that of the cells maintained with fetal bovine serum [48]. In addition, synovial cells derived from older human osteoarthritic donors could be reprogrammed to pluripotent cells in alginate culture by stimulation of BMP-2 or BMP-7 in dexamethasone- and serum-free conditions [49]. These results showed that SM has a therapeutic potential for treatment of chondral defects using *in vitro* experiments, since human autologous serum increased the proliferative potential of SM MSCs through platelet-derived growth factors signaling activation [48]. MSC-like cells from SM can be found in healthy and OA cartilage [29, 49].

**Figure 1:**
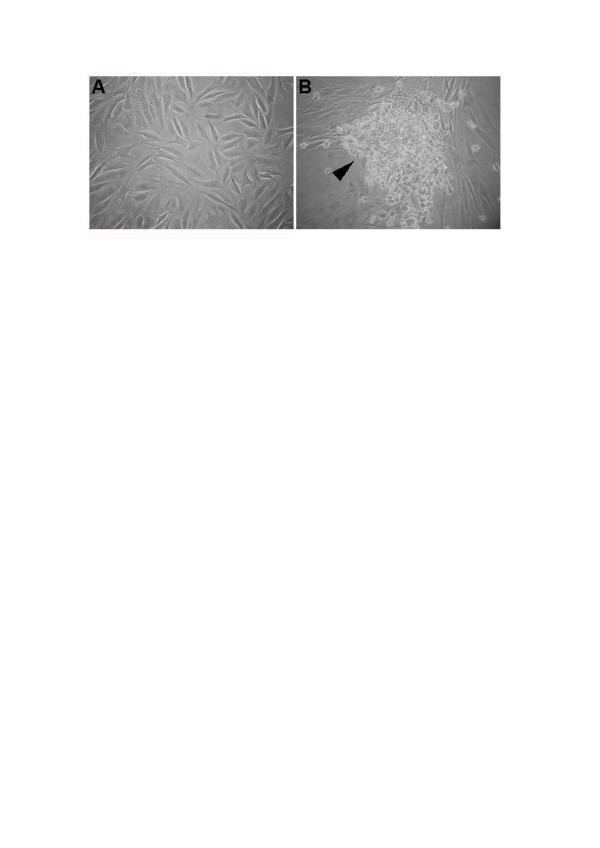
**Morphological aspects of synovial fluid mesenchymal stem cells isolated from (A) healthy persons and (B) patients with osteoarthritis.** Cell cluster (arrowhead) is observed in synovial fluid mesenchymal stem cells from patients with osteoarthritis.

BM cells imbedded in growth factors such as TGF-β, BMP, and insulin-like growth factors (IGFs) have an important role in the repair of cartilage defects [50]. Members of the BMP family, mainly BMP-7 and IGF-1, have demonstrated *in vitro* ability to stimulate chondrogenesis [23]. The problem remains in the complexity of the signaling pathways involved in chondrogenesis stimulated by cell-to-cell contact [23]. The chemokine profile of healthy and arthritic SF could contribute to the recruitment of human mesenchymal progenitor from the subchondral bone [51]. Human SF from healthy persons and OA and rheumatoid arthritis donors contains different levels of chemokines such as CCL22, Ccl27, CXCL5, and CXCL12, inhibiting migration of human subchondral mesenchymal progenitors. However, other chemokines found in SF, such as CCL2, CCL24, and CXCL7, had no effect on the attraction of mesenchymal progenitor cells [51]. The number of MSCs recruited by SF from rheumatoid arthritis patients is lower than from OA or normal donors, suggesting that the chemotactic factors contribute to the attraction of progenitors [50]. We have observed distinct morphological aspects of cells derived from SF of healthy persons and OA patients (Figure 1). It was reported that SF MSC levels in normal knee joints increased sevenfold in early OA [7]. SF MSCs probably participate in homeostasis, remodeling, and tissue repair through the replacement of cells. We can speculate that these cells are liable to re-establish the imbalance between OA catabolism and joint anabolism.

## Conclusions

SF seems to have a role in attracting MSCs whether from BM or other sources on the synovial joint. This role is crucial for maintaining joint homeostasis. Exploring these mechanisms seems to be the way to find a potential treatment for cartilage degeneration. Additional improvement should be pursued to achieve more efficient therapy for patients with OA. Moreover, the anti-proliferative and anti-migratory function in SF MSCs in patients with OA could be used to reduce cartilage destruction by SF MSCs. Cartilage bioengineering involves cell differentiation and extracellular matrix synthesis in a stratified conformation that replicates native cartilage. We believe that more basic, translational, and clinical studies involving SF MSCs will lead to improvements in OA treatment.

## Abbreviations

AT: Adipose tissue
BM: Bone marrow
BMP: Bone morphogenetic protein
HA: Hyaluronic acid
IGF: Insulin-like growth factor
IL: Interleukin
MSC: Mesenchymal stem cell
OA: Osteoarthritis
SF: Synovial fluid
SM: Synovial membrane
TGF-β: Transforming growth factor-beta
UDP: Uridine diphosphate
UDPGD: Uridine diphosphate glucose dehydrogenase.
